# Sensory Tricks in Primary Cervical Dystonia Depend on Visuotactile Temporal Discrimination

**DOI:** 10.1002/mds.25305

**Published:** 2013-01-02

**Authors:** Georg Kägi, Petra Katschnig, Mirta Fiorio, Michele Tinazzi, Diane Ruge, John Rothwell, Kailash P Bhatia

**Affiliations:** 1 Sobell Department of Motor Neuroscience and Movement Disorders, Institute of NeurologyQueen Square, London, United Kingdom; 2 Department of Neurology, Kantonsspital St. GallenSt. Gallen, Switzerland; 3 Department of Neurology, Medical University of GrazGraz, Austria; 4 Department of Neurological, Neuropsychological, Morphological and Movement Sciences, University of VeronaVerona, Italy; 5 Neurology Unit, Borgo Trento HospitalVerona, Italy

**Keywords:** primary adult-onset cervical dystonia, temporal sensory discrimination, sensory trick, geste antagoniste

## Abstract

A characteristic feature of primary cervical dystonia is the presence of “sensory tricks” as well as the impairment of temporal and spatial sensory discrimination on formal testing. The aim of the present study was to test whether the amount of improvement of abnormal head deviation due to a sensory trick is associated with different performance of temporal sensory discrimination in patients with cervical dystonia. We recruited 32 patients with cervical dystonia. Dystonia severity was assessed using the Toronto Western Spasmodic Torticollis Rating Scale. Patients were rated according to clinical improvement to a sensory trick and assigned to 1 of the following groups: (1) no improvement (n = 6), (2) partial improvement (n = 17), (3) complete improvement (n = 9). Temporal discrimination thresholds were assessed for visual, tactile, and visuotactile modalities. Disease duration was shorter (*P* = .026) and dystonia severity lower (*P* = .033) in the group with complete improvement to sensory tricks compared with the group with partial improvement to sensory tricks. A significant effect for group and modality and a significant interaction between group × modality were found, with lower visuotactile discrimination thresholds in the group with complete improvement to sensory tricks compared with the other groups. In primary cervical dystonia, a complete resolution of dystonia during a sensory trick is associated with better visuotactile discrimination and shorter disease duration compared with patients with less effective sensory tricks, which may reflect progressive loss of adaptive mechanisms to basal ganglia dysfunction. © 2013 *Movement* Disorder Society

Dystonia is characterized by sustained involuntary muscle contractions causing twisting and abnormal posturing.^1^ Adult-onset primary cervical dystonia (AOPCD) is the most common form of adult-onset focal dystonia.^2^ The incidence is sex- and age dependent. Women are affected 1.5–1.9 times more often than men, and in both men and women the peak incidence is in the fifth decade. In addition to dysfunction of the basal ganglia, other brain regions such as the cerebellum, thalamus, cerebral cortex, and midbrain/brain stem may be involved in the etiology of dystonia.^3^ A characteristic feature of primary dystonia and especially of adult-onset primary cervical dystonia is the improvement of dystonic contractions during a sensory maneuver (sensory trick), which can be observed in about three quarters of patients.^4^,^5^ In cervical dystonia, the sensory maneuver usually consists of touching the face contra- and/or ipsilateral to the direction of head rotation, or sometimes even imagination can reduce or abolish involuntary muscle activity.^4^,^6^,^7^ The presence of this phenomenon and that sensory abnormalities can be present led to the hypothesis that dystonia is not only a motor but also a sensory disorder.^8^,^9^ In fact, patients with adult-onset primary focal dystonia have impaired spatial^10^ and temporal sensory discrimination,^11^ as indicated by increased thresholds on standardized sensory testing. Furthermore, there is good evidence from functional imaging^12^ and from electrophysiological^13^ studies to support this hypothesis. In the former study, dramatic disorganization in the primary sensory cortex of nondystonic hand representation was found in all patients, and its amount paralleled the severity of dystonic limb motor impairment.^12^ The latter study, investigating central sensory integration in dystonia using somatosensory-evoked potentials, showed that the inhibitory integration of afferent inputs, mainly proprioceptive, coming from adjacent body parts is abnormal.^13^

On the basis of the findings that a successful sensory trick requires sensory input to be processed and that sensory discrimination can be impaired in primary dystonia, we hypothesized that the more the sensory processing is impaired, the less successful the sensory trick will be. The aim of this study was to test this hypothesis on patients with adult-onset primary cervical dystonia, by measuring temporal sensory (visual, tactile, visuotactile) discrimination.

## Patients and Methods

We recruited patients with AOPCD who were attending the movement disorder or botulinum toxin clinics at the National Hospital for Neurology and Neurosurgery, London, as well as healthy controls. After giving written consent, eligible patients were clinically examined by an experienced movement disorders specialist (G.K.). Participants with clinical evidence of polyneuropathy or carpal tunnel syndrome were excluded. Dystonia severity was quantified with the Toronto Western Spasmodic Torticollis Rating Scale (TWSTRS), which is divided into 3 subscales. Subscale I assesses motor severity, with a maximum score of 35; subscale II assesses disability, with a maximum score of 30; and subscale III assesses pain severity and its disability, with a maximum score of 20.^14^,^15^ To assess the effect of a sensory trick, the patients were asked to apply the most effective sensory trick in their experience, which was rated as follows: 0 points for an absent response, 1 point for some effect, and 2 points for complete resolution of cervical dystonia during the sensory trick. The patients were assigned to 1 of the 3 groups according to the rating of the sensory trick (no sensory trick, partial sensory trick, or complete sensory trick).

Temporal discrimination thresholds were examined with pairs of tactile, visual, and tactile/visual (crossmodal) stimuli. Square-wave electrical pulses, which were delivered by a constant current stimulator (DS3, Digitimer Ltd.) through surface skin electrodes (4 mm in diameter) were used for tactile stimulation. The electrodes were placed on the index and middle fingers of both hands. The anode was 1.5 cm distal from the cathode. The intensity of tactile stimulation for each subject and each finger was determined by delivering a series of stimuli with increasing intensity from 2 milliamperes (mA) in steps of 1 mA. The minimal intensity at which electric stimuli were perceived in 10 of 10 stimuli was used in the experimental test. Care was taken that stimuli did not induce pain or discomfort.

Visual stimuli were delivered through light-emitting diodes (LEDs) positioned on a black table. Subjects' hands were positioned near the LEDs. Subjects were asked to look at the fixation point in the middle between the LEDs throughout each trial. Both visual and tactile stimuli lasted 5 ms. The experimental test was delivered in 6 combinations of stimulation: 2 tactile (left and right), 2 visual (left and right), and 2 crossmodal (visuotactile left and visuotactile right). The order in which the 6 combinations of stimuli were presented was randomized across the subjects. Each combination of stimuli was performed in 4 separate blocks. In the first trial of each block, pairs of simultaneous stimuli (interstimulus interval [ISI] = 0 ms) were delivered. In subsequent trials, ISIs were progressively increased in steps of 10 ms. In each block trials with simultaneous stimuli were interspersed to check for attention.

The temporal discrimination threshold was considered the first of 3 consecutive ISIs during which subjects recognized the stimuli as asynchronous. In addition, subjects were asked to judge which stimulus preceded (or followed) the other. The first of 3 consecutive ISIs during which subjects also reported correctly the temporal order in a pair of stimuli was termed temporal order judgment.

### Statistical Analysis

Data were analyzed using SPSS Statistical Package for Windows version 14.0 and R statistical software (version 2.9.0). Data are presented as mean and standard deviation of the mean for age, age at disease onset, disease duration, TWSTRS score, TWSTRS subscores I–III, visual-, tactile-, and crossmodal temporal discrimination thresholds, and temporal order judgments. One-way ANOVA with post hoc Bonferroni test was applied with the sensory trick group as factor and age, age of onset, disease duration, TWSTRS score, and subscores I–III as dependent variables. Temporal discrimination thresholds (visual, tactile, crossmodal) were first analyzed using a 3-way ANOVA model with time (in milliseconds) as the dependent variable and sensory trick group (no, partial, complete sensory trick), modality (visual, tactile, crossmodal), and task (temporal discrimination threshold, temporal order judgment) as explanatory variables (fixed factors), including an interaction term between sensory trick group and modality. Post hoc pairwise comparisons between the fixed factors were performed using Tukey's procedure. *P* < .05 was considered significant. In primary adult-onset primary cervical dystonia, existing literature suggests that sensory discrimination thresholds depend on age but less on disease severity or disease duration. Therefore, Pearson's correlation coefficients were calculated to characterize and test (without adjustment for multiple testing) these associations.

## Results

Thirty-two patients (24 women, 8 men) with adult-onset primary cervical dystonia were included. The mean age of patients was 56.4 ± 9.9 years (women, 56.3 ± 9 years; men, 57 ± 13.1 years), mean age of onset was 44 ± 11.4 years (women, 44.4 ± 9.6 years; men, 42.5 ± 16 years), and mean disease duration was 12.5 ± 8.5 years (women, 11.8 ± 8.5 years; men, 14.5 ±8.9 years). Eighty-four percent of our study population was right-handed. The mean TWSTRS score was 24.7 ± 9.3 (women, 25.9 ± 9.6; men, 21.2 ± 8.2). An improvement in a sensory maneuver was present in 20 of 24 female patients (83%) versus 6 of 8 male patients (75%). Mean temporal discrimination thresholds were: visual, 53.2 ± 15.7 ms (women, 54.1 ± 15.5 ms; men, 50.4 ± 17.2 ms); tactile, 85.7 ± 38.5 ms (women, 90.3 ± 41.5 ms; men, 71.9 ± 24.6 ms); and crossmodal (visual/tactile), 134.5 ± 38.7 ms (women, 135.4 ± 32.1 ms; men, 131.8 ± 56.7 ms). Mean temporal order judgments were: visual, 55.3 ± 16.2 ms (women, 56.5 ± 16.4 ms; men, 51.7 ± 16.2 ms); tactile, 101.2 ± 46.6 ms (women, 109.1 ± 49.2 ms; men, 77.6 ± 28.5 ms); and crossmodal (visual/tactile), 141.3 ± 39.3 ms (women, 142.7 ± 33.2 ms; men, 136.9 ± 56.7 ms). No significant effect was found for the factor sex for either of the evaluated variables. Six patients with adult-onset primary cervical dystonia (19%) were allocated to the no sensory trick group, 17 patients (53%) to the partial sensory trick group, and 9 patients (28%) to the complete sensory trick group. As presented in Table 1, significant effects were found for the sensory trick group and disease duration (*F* = 4.6, *P* = .018), with a significantly (*P* = .026) shorter disease duration in patients with a complete sensory trick compared with patients with a partial sensory trick in the post hoc analysis and for the TWSTRS score (*F* = 3.8, *P* = .035) and subscore III (*F* = 5.3, *P* = .009) with significantly lower scores in patients with a complete sensory trick compared with patients with a partial sensory trick in the post hoc analysis (*P* = .033 and *P* = .009, respectively). No significant effects were found for age, age of onset, or TWSTRS subscores I and II.

**TABLE 1:** Clinical and demographic data according to the sensory trick

	Patients					
				
	No ST	Partial ST	Complete ST	No ST vs partial ST *P*	No ST vs complete ST *P*	Partial ST vs complete ST *P*
Number (%)	6 (19)	17 (53)	9 (28)	—	—	—
Female/male	4/2	13/4	7/2	1	1	1
Age	58.2 ± 10.1	55.7 ± 9.7	56.8 ± 11.3	1	1	1
Age of onset	42.5 ± 8.5	40.8 ± 11.5	50.9 ± 10.9	1	.461	.094
Disease duration	15.7 ± 10.5	14.8 ± 8.5	5.9 ± 1.4	1	.067	.**026**
TWSTRS total	23.6 ± 12.3	28.3 ± 8.5	18.7 ± 5.3	.767	.862	.**033**
TWSTRS subscore I	12.2 ± 3.1	12.1 ± 2.4	10.2 ± 3.6	1	.639	.405
TWSTRS subscore II	6.7 ± 4	8.9 ± 3.9	6.6 ± 4.7	.783	1	.530
TWSTRS subscore III	6.3 ± 5.8	7.4 ± 4.3	1.9 ± 2.2	1	.170	.**009**

All values are means with standard deviation unless stated otherwise.

*P* < .05 (ANOVA with post hoc Bonferroni) in bold.

The 3-way ANOVA model showed significant effects for the sensory trick group (*F* = 6.2, *P* = .002) and modality (*F* = 101.8, *P* < .001) but not for task. Therefore, temporal discrimination thresholds and temporal order judgments were averaged to further test the interaction sensory trick group × modality, which was significant (*F* = 2.5, *P* = .046). Post hoc pairwise analysis of the interaction between sensory trick group and modality showed significant differences in the crossmodal paradigm between the complete sensory trick and no sensory trick groups (*P* = .049) as well as between the complete sensory trick and partial sensory trick groups (*P* = .008), whereas no significant difference was found between the no sensory trick and the partial sensory trick groups. Post hoc analysis did not show significant differences between the sensory trick groups either for the visual or for the tactile paradigms. Pairwise post hoc analysis between different modalities within the same sensory trick group throughout showed significant differences, except for the tactile versus the crossmodal paradigm in the group without sensory trick and the visual versus tactile paradigm in the complete sensory trick group (Table 2).

**TABLE 2:** Electrophysiological results according to the sensory trick

Sensory trick		Visual (ms)	Tactile (ms)	Crossmodal (ms)	Row mean (ms)
Absent	tdt	49.9 ± 13.4	97.2 ± 46.5	147.7 ± 24.4	98.3 ± 50.5
	toj	53.9 ± 13.2	121.4 ± 52.5	152.9 ± 31.4	109.4 ± 54.4
	Mean	51.9 ± 12.9	109.3 ± 48.9	150.3 ± 27.0	103.8 ± 18.2
Partial	tdt	54.0 ± 15.2	89.7 ± 41.1	144.5 ± 42.8	96.0 ± 51.1
	toj	54.8 ± 15.4	101.2 ± 46.5	150.6 ± 43.7	102.2 ± 54.2
	Mean	54.4 ± 15.1	95.4 ± 43.6	147.5 ± 42.7	99.1 ± 16.2
Complete	tdt	53.8 ± 19.3	70.5 ± 24.6	106.8 ± 23.9	77.0 ± 31.4
	toj	57.2 ± 20.7	87.7 ± 43.2	115.9 ± 23.5	87.0 ± 38.4
	Mean	55.5 ± 19.5	79.1 ± 35.3	111.4 ± 23.5	82.0 ± 8.2

All values are means with standard deviation.

ms, milliseconds; tdt, temporal discrimination threshold; toj, temporal order judgment.

Among all patients, age correlated with visual temporal discrimination (temporal discrimination threshold: *r* = 0.441, *P* = .011; temporal order judgment: *r* = 0.406, *P* = .021) and with tactile temporal discrimination without reaching the level of significance (temporal discrimination threshold: *r* = 0.339, *P* = 0.058; temporal order judgment: *r* = 0.343, *P* = .054), but not with crossmodal temporal discrimination. Motor disease severity, expressed by TWSTRS subscore I, and disease duration did not correlate with the temporal discrimination thresholds.

## Discussion

This psychophysical study on sensory tricks in patients with adult-onset primary cervical dystonia revealed relevant findings that require further discussion.

In this study, patients with complete improvement to a sensory maneuver had shorter disease duration and a lower TWSTRS score compared with patients with only partially effective sensory tricks, whereas patients' age did not differ between the 3 groups (no, partial, complete sensory trick). The finding that patients with a short disease duration experienced a better effect of a sensory maneuver has been described in different types of focal dystonia, such as cervical dystonia,^6^,^16^,^17^ writer's cramp,^18^ and blepharospasm.^19^ The relationship of disease severity and effectiveness of the sensory trick found in this study has also been found in other studies,^6^,^17^ whereas this relationship was not significant in another recent study on sensory tricks by Martino et al.^5^ Because only the latter study applied the Burke-Fahn-Marsden score compared with the TWSTRS in the other studies (our study included), differences in the range of scores and in the validity might partly account for this discrepancy. Another important factor seems to be the differentiation between forcible (pushing against the direction of head deviation) and classic (only light touch) sensory tricks. Ochudlo et al^17^ analyzed the relationship between clinical data in patients with forcible and classic sensory tricks separately. They found that forcible sensory tricks were associated with higher disease severity on the TWSTRS, whereas the opposite was true for patients with classic sensory tricks. The fact that we found less severe disease only in the complete sensory trick group compared with the partial and no sensory trick groups seems to provide evidence that a sensory maneuver that releases dystonia completely represents a classic sensory trick, whereas partial sensory tricks seem to include more forcible sensory tricks and therefore, from a pathophysiological point of view, are closer to the group without sensory tricks than to the group with complete sensory tricks. In the study by Martino et al,^5^ both forcible and classic sensory tricks were included.

**FIG. 1:**
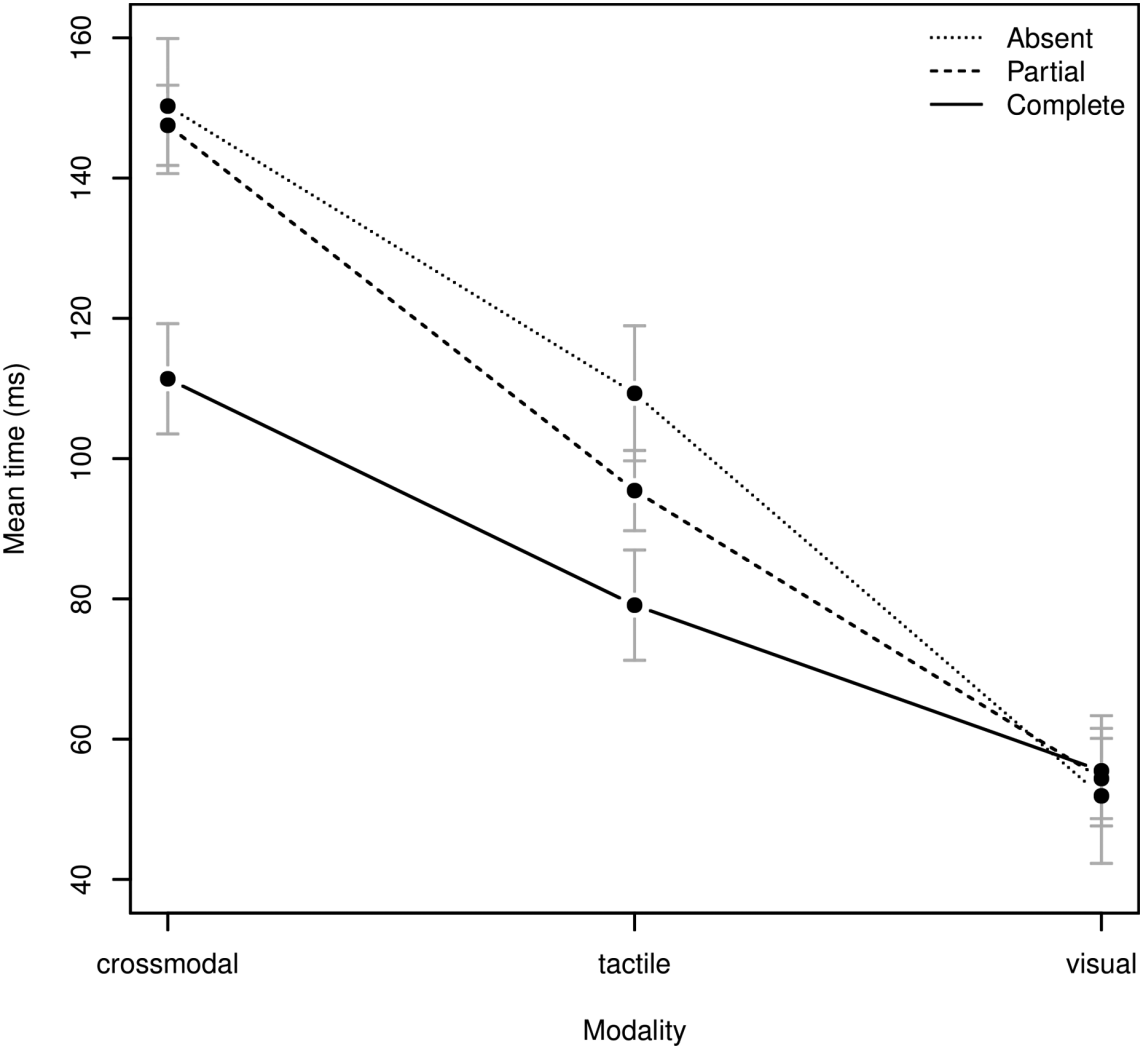
Interaction plot with standard error of the estimates showing the 2-way interactions between modality and sensory trick after averaging tdt and toj (absent, no sensory trick group; partial, partial sensory trick group; complete, complete sensory trick group).

One interesting result of the present study is that patients with a complete sensory trick performed significantly better in the crossmodal temporal discrimination task, as expressed by lower thresholds, compared to patients with only a partial or absent effect to a sensory trick. It is particularly interesting that only the crossmodal task revealed differences between the groups selected by the effect of a sensory maneuver, whereas the unimodal tasks did not (see Fig. 1). This may relate to our finding that sensory tricks and sensory discrimination both deteriorate with increasing duration of disease. In contrast, when taken across the whole group of patients, unimodal temporal discrimination tasks were correlated with age (visual and a trend for tactile), but no correlation was found with disease severity and disease duration. In this context it should be noted that there is considerable variation in disease progression. Although loss of the sensory trick with longer disease duration is true for most patients with adult-onset primary cervical dystonia, there are also patients who never lose or never experience the effect.

The results of a number of other studies have consistently shown a relationship between processing of sensory input and sensory tricks. One study investigated patients with blepharospasm and found an association between the presence of sensory tricks and normal prepulse inhibition of the blink reflex.^20^ In fact, in those experiments prepulse effects depended on activation of trigeminal afferents, consistent with the idea that abnormal processing of that sensory input is associated with the absence of the sensory trick. Another fMRI study on patients with writer's cramp found increased activation of basal ganglia nuclei and various cortical areas (bilateral visual areas, contralateral anterior insula, and ipsilateral parietal cortex [intraparietal sulcus]) during discrimination of tactile stimuli. Interestingly, the effect was smaller in patients with a longer duration of symptoms but had no correlation with clinical severity.^18^ One possible explanation is that overactivation early in the course of the disease represents recruitment of a compensatory mechanism that declines in effectiveness over the course of the disease because of abnormal processing of sensory input. Finally, Naumann et al^21^ noted in a PET study that performance of a sensory trick that normalized head position in patients with torticollis increased activity in the ipsilateral (to the direction of head turn) superior and inferior parietal lobules and reduced activity of the contralateral supplementary motor area and primary sensorimotor cortex. The authors explained this particular activation of the ipsilateral parietal cortex, which integrates different sensory modalities to form a cognitive representation of space, with a disturbed body scheme in patients with adult-onset primary cervical dystonia. They speculated that the perceptual imbalance induced by the sensory trick displaced the egocentric midvertical reference toward the opposite side and increased ipsilateral parietal activity.

These observations from functional imaging studies showing that the parietal cortex, as an important center of multimodal sensory integration, is hyperactive during the execution of a sensory maneuver is well in line with our findings of better performance (lower temporal crossmodal [visual/tactile] discrimination thresholds) in patients with a complete sensory trick compared to patients with less effective or absent sensory tricks. This means that parietal function (multimodal sensory integration) decreases with longer disease duration. As shown in the prepulse blink reflex inhibition or in the fMRI experiments, the progressive increase in temporal crossmodal discrimination thresholds may reflect progressive loss of cortical adaptive mechanisms to the basal ganglia dysfunction of adult-onset primary cervical dystonia. Our results provide evidence that patient assignment according to their sensory trick represents a different selection from a pathophysiological point of view where the functional integrity of the parietal cortex is important, which is reflected by the crossmodal sensory discrimination thresholds.

In summary, this study confirms that in adult-onset primary cervical dystonia, sensory tricks are more prevalent in patients with short disease duration and less severe disease expressed by low TWSTRS score, whereas it does not depend on the patient's age. This could be explained by intact sensory afferents in early disease stages that get impaired the longer disease progresses. On a cortical (parietal) level, functionally intact adaptive mechanisms to the basal ganglia dysfunction seem to be a premise for the presence of a good clinical effect to a sensory maneuver, because with its loss, crossmodal (visuo/tactile) temporal discrimination thresholds increase.

## References

[1] Fahn S (1988). Concept and classification of dystonia. Adv Neurol.

[2] Nutt JG, Muenter MD, Aronson A, Kurland LT, Melton LJ (1988). Epidemiology of focal and generalized dystonia in Rochester, Minnesota. Mov Disord.

[3] Neychev VK, Gross RE, Lehéricy S, Hess EJ, Jinnah HA (2011). The functional neuroanatomy of dystonia. Neurobiol Dis.

[4] Deuschl G, Heinen F, Kleedorfer B, Wagner M, Lucking CH, Poewe W (1992). Clinical and polymyographic investigation of spasmodic torticollis. J Neurol.

[5] Martino D, Liuzzi D, Macerollo A, Aniello MS, Livrea P, Defazio G (2010). The phenomenology of the geste antagoniste in primary blepharospasm and cervical dystonia. Mov Disord.

[6] Muller J, Wissel J, Masuhr F, Ebersbach G, Wenning GK, Poewe W (2001). Clinical characteristics of the geste antagoniste in cervical dystonia. J Neurol.

[7] Greene PE, Bressman S (1998). Exteroceptive and interoceptive stimuli in dystonia. Mov Disord.

[8] Hallett M (1995). Is dystonia a sensory disorder?. Ann Neurol.

[9] Stamelou M, Edwards MJ, Hallett M, Bhatia KP (2012). The non-motor syndrome of primary dystonia: clinical and pathophysiological implications. Brain.

[10] Bara-Jimenez W, Shelton P, Hallett M (2000). Spatial discrimination is abnormal in focal hand dystonia. Neurology.

[11] Tinazzi M, Fiorio M, Bertolasi L, Aglioti SM (2004). Timing of tactile and visuo-tactile events is impaired in patients with cervical dystonia. J Neurol.

[12] Meunier S, Garnero L, Ducorps A (2001). Human brain mapping in dystonia reveals both endophenotypic traits and adaptive reorganization. Ann Neurol.

[13] Tinazzi M, Priori A, Bertolasi L, Frasson E, Mauguiere F, Fiaschi A (2000). Abnormal central integration of a dual somatosensory input in dystonia. Evidence for sensory overflow. Brain.

[14] Comella CL, Stebbins GT, Goetz CG, Chmura TA, Bressman SB, Lang AE (1997). Teaching tape for the motor section of the Toronto Western Spasmodic Torticollis Scale. Mov Disord.

[15] Consky E, Lang A (1994). Therapy with Botulinum Toxin.

[16] Dauer WT, Burke RE, Greene P, Fahn S (1998). Current concepts on the clinical features, aetiology and management of idiopathic cervical dystonia. Brain.

[17] Ochudlo S, Drzyzga K, Drzyzga LR, Opala G (2007). Various patterns of gestes antagonistes in cervical dystonia. Parkinsonism Relat Disord.

[18] Peller M, Zeuner KE, Munchau A (2006). The basal ganglia are hyperactive during the discrimination of tactile stimuli in writer's cramp. Brain.

[19] Gomez-Wong E, Marti MJ, Tolosa E, Valls-Sole J (1998). Sensory modulation of the blink reflex in patients with blepharospasm. Arch Neurol.

[20] Gomez-Wong E, Marti MJ, Cossu G, Fabregat N, Tolosa ES, Valls-Sole J (1998). The ‘geste antagonistique’ induces transient modulation of the blink reflex in human patients with blepharospasm. Neurosci Lett.

[21] Naumann M, Magyar-Lehmann S, Reiners K, Erbguth F, Leenders KL (2000). Sensory tricks in cervical dystonia: perceptual dysbalance of parietal cortex modulates frontal motor programming. Ann Neurol.

